# Feasibility and outcomes of robotic sphincter-preserving surgery for rectal cancer after neoadjuvant treatment in patients with preexisting colostomy

**DOI:** 10.1007/s10151-024-02980-w

**Published:** 2024-08-13

**Authors:** H. Nozawa, A. Sakamoto, K. Murono, K. Sasaki, S. Emoto, S. Ishihara

**Affiliations:** Department of Surgical Oncology, The University of Tokyo, 7-3-1 Hongo, Bunkyo-Ku, Tokyo, 113-8655 Japan

**Keywords:** Robotic surgery, Sphincter preserving, Rectal cancer, Preexisting stoma, Outcomes

## Abstract

**Background:**

Diverting colostomy followed by neoadjuvant treatment is a treatment of choice for obstructive rectal cancer. Such patients may be treated via a robotic approach with several advantages over conventional laparoscopic surgery. Conversely, the existing stoma may interfere with the optimal trocar position and thus affect the quality of robotic surgery. Moreover, the console surgeon does not face the patient, which may endanger the stoma.

**Methods:**

Patients with rectal cancer who underwent sphincter-preserving surgery were retrospectively investigated using a robotic platform after neoadjuvant treatment at our hospital. Based on pretreatment stoma creation, patients were divided into the NS (those without a stoma) and S groups (patients with a stoma). Baseline characteristics, types of neoadjuvant treatment, short-term surgical outcomes, postoperative anorectal manometric data, and survival were compared between the groups.

**Results:**

The NS and S groups comprised 65 and 9 patients, respectively. Conversion to laparotomy was required in three patients in the NS group. The S group required a longer console time than the NS group (median: 367 vs. 253 min, respectively, *p* = 0.038); however, no difference was observed in the total operative time (*p* = 0.15) and blood loss (*p* = 0.70). Postoperative complication rates, anorectal function, and oncological outcomes were similar between the groups.

**Conclusions:**

Although console time was longer in patients with a stoma, robotic surgery could be performed safely like in those without a stoma after neoadjuvant treatment.

## Introduction

Rectal cancer is one of the most common malignancies worldwide, with approximately 340,000 annual deaths in 2020 [1]. The burden of rectal cancer is increasing among populations younger than 50 years [2]. Over the past 3 decades, several randomized controlled trials (RCTs) have demonstrated that neoadjuvant chemoradiotherapy (NACRT) followed by total mesorectal excision (TME) has been widely adopted to increase the pathological complete remission (pCR) rate and to decrease the local recurrence rate [3, 4]. Thus, NACRT is recommended as a standard treatment sequence for patients with locally advanced lower rectal cancer [5, 6]. In addition, neoadjuvant chemotherapy (NAC) using chemotherapeutic drugs and biologics conferred similar oncological benefits and resulted in fewer treatment-related complications than NACRT in patients with rectal cancer, as shown by the PROSPECT trial and other studies [7–9]. Moreover, the addition of preoperative chemotherapy pre- or post-NACRT achieved a higher pCR rate [10], causing a paradigm shift to the concept of total neoadjuvant therapy [5].

Approximately 10%–20% of colorectal cancers are obstructive at diagnosis [11, 12]. Several obstructive cases require urgent decompression by placing a self-expandable metallic stent (SEMS), transanal decompression tube, or diverting stoma unless the primary tumor is immediately resected. Among these choices, the World Society of Emergency Surgery Guidelines state that loop colostomy creation is preferable when preoperative therapies are predicted in rectal cancer [13]. SEMS may have negative oncologic impacts, such as increased circulating cancer cells and aggressive pathological characteristics, even in clinically successful patients without perforation [14]. The safety of neoadjuvant treatment after SEMS placement in obstructive rectal cancer is also a major concern [14]. Moreover, the European Society of Gastrointestinal Endoscopy indicates a time interval of approximately 2 weeks between stent insertion and tumor resection as a bridge to elective surgery [15]. Meanwhile, a multicenter study in Japan showed that transanal decompression tube placement caused more frequent complications in patients with malignant large bowel obstruction than SEMS [16]; this report also showed fewer local recurrences in patients treated by SEMS than transanal tube placement [16]. Based on these discussions, a diverting colostomy for obstructive rectal cancer was usually created, and neoadjuvant treatment such as NAC and/or NACRT was initiated at our hospital.

With the advent of minimally invasive surgery, many patients with obstructive rectal cancer have undergone rectal surgery via a robotic approach. Compared with conventional laparoscopic surgery, the robot-assisted surgical platform has several characteristics, such as three-dimensional vision, a greater range of motion and precision of instruments, and better ergonomics. Conversely, a preexisting stoma may interfere with the optimal trocar position and thus affect the quality of robotic surgery. In addition, the stoma may be endangered because the patient is out of view of the surgeon facing the console during surgery. These may cause poor surgical outcomes of robotic surgery such as prolonged operative duration, a high likelihood of complications, a high rate of positive circumferential resection margins, and inferior long-term survivals.

Given the above, the surgical outcomes of robotic rectal surgery after neoadjuvant treatment were investigated in patients with a preexisting stoma by comparing them with those in ostomy-free patients.

## Materials and methods

### Patients

A retrospective review of consecutive patients with rectal cancer who underwent sphincter-preserving surgery after neoadjuvant treatment at our department was carried out between February 2018 and December 2023. Among them, patients who underwent surgical resection of disease of other organs were excluded, whereas patients who underwent resection of organs directly invaded by the primary tumor were included. Patients who underwent simultaneous resection of other colonic segments or total proctocolectomy for colitis-associated neoplasms were also excluded.

Before neoadjuvant treatments, a diverting colostomy was created in patients with obstructive rectal cancer, e.g., endoscopically untraversable tumor with/without obstructive symptoms or future obstruction caused by chemotherapy and/or radiotherapy-associated edema and fibrosis. Based on the pretreatment stoma creation, patients were divided into the NS (those without a stoma) and S groups (patients with a stoma).

This study was approved by the University of Tokyo Institutional Review Board (3252–17). Written informed consent was obtained from all patients, and we also provided the opportunity to opt out from inclusion in this study.

### Neoadjuvant and adjuvant treatments

NACRT was administered to patients with lower rectal cancer at the cT3-4 and/or N + stage without distant metastasis or with oligo-metastasis, as described in our previous reports [17]. In brief, the total dose of preoperative radiotherapy was 50.4 Gy over 6 weeks (28 fractions). The clinical target volume included the primary tumor, anus, and regional lymph nodes such as those around the inferior mesenteric and internal iliac middle rectal vessels and obturator foramen. Tegafur-uracil with leucovorin was administered concomitantly as a long-course CRT. Biweekly irinotecan was additionally infused four times (TEGAFIRI regimen) in patients enrolled in a phase I/II study [18], which has been applied as a routine regimen since 2019.

NAC was administered to patients with resectable distant metastasis. A multidisciplinary team determined the regimen and duration. Targeting antibodies were concomitantly administered based on the *ras/braf* gene status of the primary rectal cancer in selected patients [19].

Moreover, NACRT plus NAC was recently administered to selected patients with lower rectal cancer with aggressive features, such as invasion to adjacent organ(s), massive nodal metastases, and extramural venous invasion, in a phase II clinical trial [20]. Typically, patients receive TEGAFIRI followed by two cycles of capecitabine plus oxaliplatin (CAPOX) [21] as consolidation chemotherapy.

Adjuvant oxaliplatin-based chemotherapy for 6 months is usually recommended for patients receiving NACRT or NAC. Four cycles of CAPOX were planned after rectal surgery in patients treated with both NACRT and NAC. However, the regimen and duration were determined partly based on patients’ preferences.

### Robotic rectal surgery

**Fig. 1:**
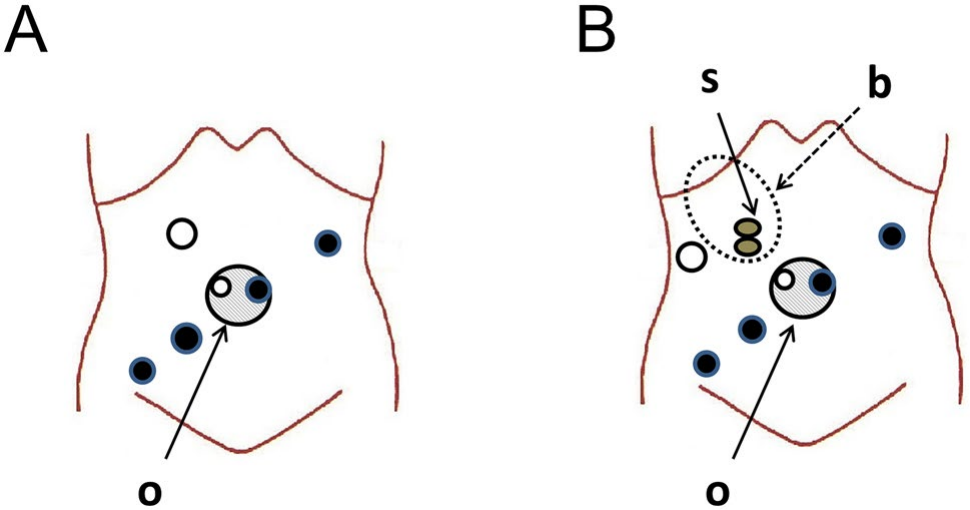
Illustrations of typical port placement for robotic rectal surgery. **A** Without a stoma. **B** With a preexisting stoma. Closed circles indicate da Vinci ports, and open circles indicate assistant ports. b: stoma bag, o: oval-shaped port device (E-Z ACCESS), s: loop ileostomy

Sphincter-preserving surgery for rectal cancer was performed using a robotic platform, da Vinci Xi (Intuitive Surgical Inc., Sunnyvale, CA). Figure 1 demonstrates the port placement for robotic rectal surgery in our department. In addition to the four da Vinci ports, two assistant ports were placed for bowel traction and suction. Among these, one da Vinci port and one assistant port were placed within an oval-shaped port device (E-Z ACCESS, Hakko Co., Ltd., Medical Device Division, Nagano, Japan) at the umbilicus. In patients with a stoma, the assistant port at the right upper quadrant was placed lateral to the stoma.

Rectal surgery was performed using a single docking of da Vinci Xi. In principle, anterior or intersphincteric resection is performed based on the TME while preserving the left colic artery, which generally enables sufficient blood perfusion without takedown of the splenic flexure [22]. Lateral lymph node dissection was selectively performed in patients who had enlarged pelvic lymph nodes (long-axis diameter ≥ 8 mm) based on pretreatment computed tomography (CT) scans [17, 18, 22]. All console surgeons involved in this study were board qualified for robotic surgery. The total operative time, console time, estimated blood loss, intraoperative blood transfusion, and open conversion were retrieved from medical charts.

### Other data collection

Data including sex; age; Eastern Cooperative Oncology Group Performance Status; body mass index (BMI); serum hemoglobin, albumin, carcinoembryonic antigen (CEA), and carbohydrate antigen (CA) 19–9 levels at the time of rectal surgery; location of the lower edge; and histology of rectal cancer were retrieved from our prospectively collected database. At our hospital, the upper normal limit is 5 ng/ml for CEA, and 37 U/ml for CA 19–9. According to the American Joint Committee on Cancer staging manual [23], the pretreatment and final stages of tumor depth (cT and ypT) and regional nodal status (cN and ypN) were determined. The tumor regression grade was classified based on the Japanese Classification of Colorectal, Appendiceal, and Anal Carcinoma, where grade 0 indicates no regression and grade 3 indicates complete regression [24]. The pathological reports were reviewed to collect the number of harvested lymph nodes and proximal, distal, and circumferential resection margins.

In-hospital postoperative complications were graded using the Clavien-Dindo classification [25]. At our department, anomanometry has been routinely conducted in patients with rectal tumors pre- and post-neoadjuvant treatment and rectal surgery using a one-channel catheter for pressure recording (GMMS-100R-SI instrument, STARMEDICAL, Inc., Tokyo, Japan) [26]. Manometric data such as maximum resting pressure (MRP) and maximum squeeze pressure (MSP) at 6 and 12 months were collected from our database for this study. Recurrence-free survival (RFS) was defined as the time interval from curative resection until the date of the first recurrence or death of any cause. Overall survival was defined as the time from the date of rectal surgery to death.

### Statistics

Categorical data were compared using the chi-square test with Yates’ correction or Fisher’s exact test. Continuous variables were compared using the Mann-Whitney U test. Survival curves were drawn using the Kaplan-Meier method and compared using the log-rank test. Statistical analyses were performed using JMP Pro 17.0.0 (SAS Institute, Cary, NC, USA). A *p* value < 0.05 was considered significant.

## Results

### Patient profile

Table 1 summarizes patient characteristics. The NS and S groups comprised 65 and 9 patients of similar age and sex distribution, respectively. The S group comprised patients with more advanced cT stage than the NS group (cT4: 56% vs 9%). No intergroup differences in other anthropometric and laboratory test data or tumor-related parameters were found preoperatively. Most patients in the NS group received NACRT alone, whereas NAC was administered to two patients (22%) and both NACRT and NAC to three patients (33%) in the S group (*p* = 0.011).

**Table 1:** Preoperative characteristics of patients

Variables		NS group (*n* = 65)	S group (*n* = 9)	*p* value
Sex	Male	43 (66%)	6 (67%)	1.00
	Female	22 (34%)	3 (33%)	
Age, years	Median (IQR)	63 (55–72)	67 (61–71)	0.56
ECOG PS	0	64 (98%)	9 (100%)	1.00
	1	1 (2%)	0 (0%)	
BMI	Median (IQR)	23 (21–25)	23 (21–25)	0.79
Hemoglobin, g/dl	Median (IQR)	12.3 (11.5–12.9)	12.4 (11.7–13.6)	0.50
Albumin, g/dl	Median (IQR)	3.9 (3.7–4.1)	3.9 (3.8–4.2)	0.73
CEA	Elevated	15 (23%)	5 (56%)	0.098
CA 19–9	Elevated	7 (11%)	2 (22%)	0.30
Histology	Differentiated	62 (95%)	9 (100%)	1.00
	Others	3 (5%)	0 (0%)	
Location of tumor	High/mid-rectum	4 (6%)	1 (11%)	0.49
	Low rectum	61 (94%)	8 (89%)	
cT stage	cT2	1 (2%)	0 (0%)	0.004
	cT3	58 (89%)	4 (44%)	
	cT4	6 (9%)	5 (56%)	
cN stage	cN0	23 (35%)	1 (11%)	0.37
	cN1	24 (37%)	3 (33%)	
	cN2	18 (28%)	5 (56%)	
Distant metastasis	Present	4 (6%)	2 (22%)	0.15
Neoadjuvant therapy	NACRT	59 (91%)	4 (45%)	0.011
	NAC	4 (6%)	2 (22%)	
	Both	2 (3%)	3 (33%)	

*IQR* interquartile range, *ECOG PS* Eastern Cooperative Oncology Group Performance Status, *BMI* body mass index, *CEA* carcinoembryonic antigen, *CA 19–9* carbohydrate antigen 19–9, *NACRT* neoadjuvant chemoradiotherapy, *NAC* neoadjuvant chemotherapy

### Short-term outcomes

Table 2 shows the short-term surgical outcomes based on the preexisting stoma. No intergroup differences in the surgical procedures and frequency of lateral lymph node dissection were observed. A diverting ileostomy was newly created in 45 patients (69%) in the NS group. Three patients (5%) in the NS group required conversion to open surgery. The S group required a longer console time than the NS group (median: 367 vs. 253 min, *p* = 0.038). The total operative time was also longer in the S group without reaching significance (median: 536 vs. 399 min, *p* = 0.15). The estimated blood loss volume was similar between the NS and S groups (*p* = 0.70).

**Table 2:** Perioperative results

Variables		NS group (*n* = 65)	S group (*n* = 9)	*p* value
Surgical procedure	High anterior resetion	0 (0%)	1 (11%)	0.44
	Low anterior resection	58 (89%)	7 (78%)	
	Intersphincteric resection	7 (11%)	1 (11%)	
Lateral lymph node dissection	No	52 (80%)	6 (67%)	0.97
	Unilateral	10 (15%)	2 (22%)	
	Bilateral	3 (5%)	1 (11%)	
Diverting stoma creation	No	20 (31%)	9 (100%)	< 0.001
	Ileostomy	45 (69%)	0 (0%)	
Conversion to laparotomy	Yes	3 (5%)	0 (0%)	1.00
Operative time, min	Median (IQR)	399 (326–532)	536 (355–686)	0.15
Console time, min	Median (IQR)	253 (191–329)	367 (236–516)	0.038
Blood loss, ml	Median (IQR)	70 (10–178)	120 (8–260)	0.70
Blood transfusion	Yes	1 (2%)	0 (0%)	1.00

*IQR* interquartile range

Table 3 displays the postoperative complications according to the preexisting stoma. The overall frequency of grade ≥ 2 complications was similar (32%–33%) between the two groups. Of note, however, one patient in the S group developed stoma prolapse exacerbation that had been reducible and underwent purse-string suture repair on day 9 after robotic surgery.

**Table 3:** Postoperative complications of grade 2 or higher

Variables	NS group (*n* = 65)	S group (*n* = 9)	*p*-value
Overall	21 (32%)	3 (33%)	1.00
Anastomotic leakage	2 (3%)	0 (0%)	1.00
Bleeding	2 (3%)	0 (0%)	1.00
Surgical site infection	2 (3%)	2 (22%)	0.070
Dysuria	2 (3%)	2 (22%)	0.070
CRBSI	1 (2%)	1 (11%)	0.23
Neuralgia	1 (2%)	0 (0%)	1.00
Thrombosis	2 (3%)	0 (0%)	1.00
Pneumonia	1 (2%)	1 (11%)	0.23
Stoma outlet obstruction^a^	7 (16%)	0 (0%)	0.59
Stoma prolapse^a^	0 (0%)	1 (11%)	0.17
Others	5 (8%)	0 (0%)	1.00

*CRBSI* catheter-related blood stream infection

^a^ The denominator was equal to the number of patients receiving a diverting stoma (45) for the NS group

### Oncological outcomes

Table 4 summarizes the oncological results. The tumor size and ypT, and ypN stages were similar between the NS and S groups. The median number of harvested nodes was 15 in both groups. No positive resection margin of primary rectal cancer was reported in either group; however, a cancer-positive node firmly fixed to the left lateral pelvic wall had to be removed with a macroscopically positive resection margin in one patient in the NS group.

**Table 4:** Pathological findings and postoperative treatment

Variables		NS group (*n* = 65)	S group (*n* = 9)	*p* value
ypT stage	―ypT1	17 (26%)	1 (11%)	0.21
	ypT2	22 (34%)	0 (0%)	
	ypT3	25 (38%)	7 (78%)	
	ypT4	1 (2%)	1 (11%)	
ypN stage	ypN0	47 (72%)	5 (56%)	0.91
	ypN1	16 (25%)	3 (33%)	
	ypN2	2 (3%)	1 (11%)	
Tumor size, mm	Median (IQR)	25 (8–35)	35 (30–44)	0.075
No. of harvested nodes	Median (IQR)	15 (11–22)	15 (13–26)	0.35
Resection margin	Positive	1 (2%)	0 (0%)	1.00
Tumor regression grade^a^	Grade 0/1	19 (29%)	4 (44%)	0.45
	Grade 2/3	46 (71%)	5 (56%)	
Adjuvant chemotherapy ^b^	None	31 (48%)	3 (33%)	0.77
	Oral 5-FU	10 (16%)	1 (11%)	
	Oxaliplatin-based	23 (36%)	5 (56%)	

*IQR* interquartile range, *5-FU* 5-fluorouracil

^a^Based on the Japanese classification of colorectal, appendiceal, and anal carcinoma

^b^The denominator was equal to the number of patients receiving curative surgery (64) for the NS group

**Fig. 2:**
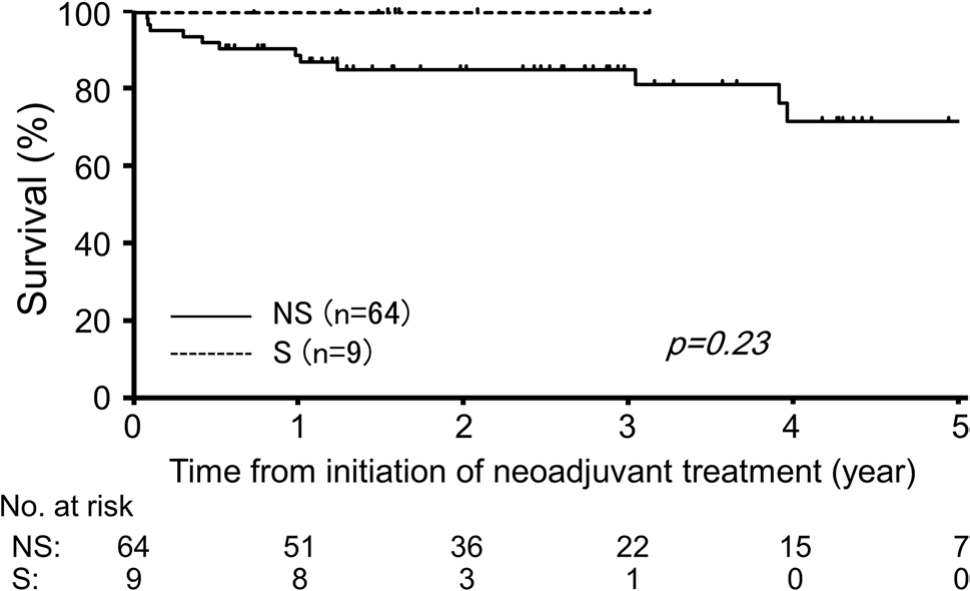
Recurrence-free survival after curative resection

Adjuvant chemotherapy was administered to 33 (52%) patients in the NS group and 6 (67%) patients in the S group (*p* = 0.77; Table 4). Kaplan-Meier survival curve analyses were performed to evaluate long-term outcomes using curatively resected cases (64 patients of the NS group and 9 patients of the S group). During the follow-up (median: 31.9 months after rectal surgery), only one patient died of cerebral hemorrhage without rectal cancer recurrence 50 months after initiating NACRT in the NS group. As shown in Fig. 2, RFS did not differ between the two groups (3-year RFS rate: 85% and 100% for the NS and S groups, respectively; *p* = 0.23)**.**

### Stoma-free rate and functional outcomes

**Fig. 3:**
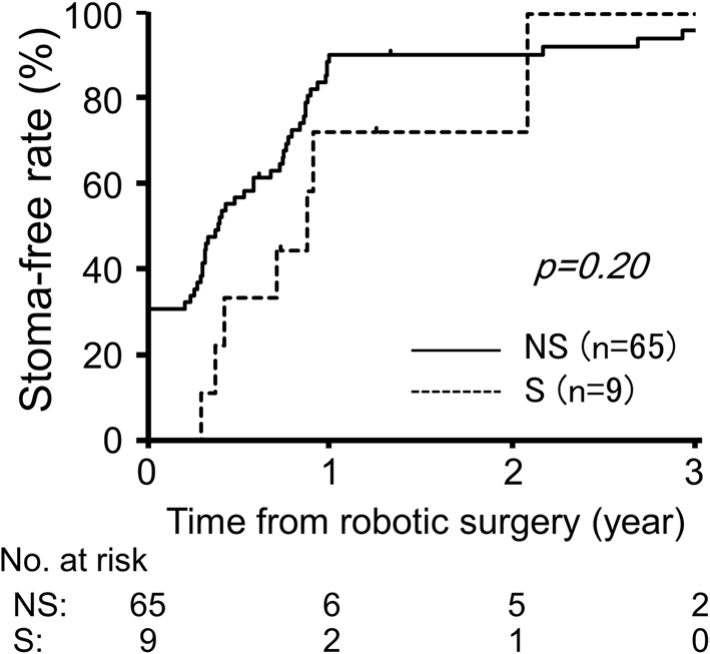
Cumulative stoma-free rate after robotic rectal surgery

To evaluate the success of sphincter-preserving surgery, the number of patients without a stoma was compared between the NS and S groups. During follow-up, stoma closure was performed in most patients with a stoma. The two groups showed comparable stoma-free rates after robotic rectal surgery (1-year rate: 90% and 72% for the NS and S groups, respectively, *p* = 0.20; Fig. 3).

**Fig. 4:**
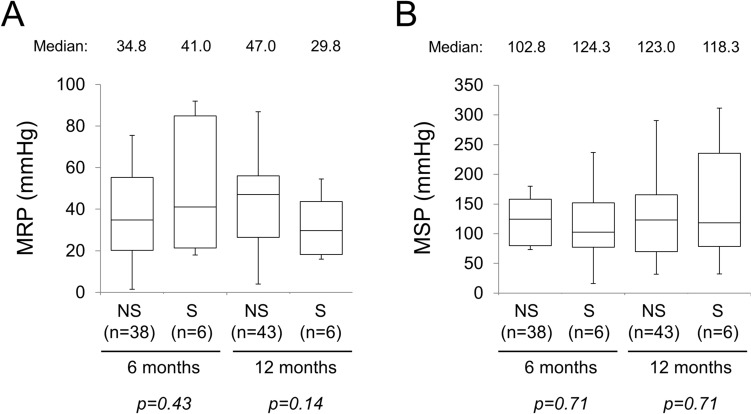
Anal function evaluated by manometry at 6 and 12 months after robotic rectal surgery. The numbers of patients evaluated are shown in parentheses. Maximum resting pressure (MRP). **A** A few high outliers were not plotted in the NS group. **B** Maximum squeeze pressure (MSP). A few high outliers were not plotted in the NS group

Because several patients prioritized adjuvant chemotherapy and/or treatment for recurrent diseases over stoma closure, anorectal functions were compared by manometry in available patients. Figure 4A shows comparative data on MRP between the NS and S groups. No significant intergroup differences were observed at 6 and 12 months after robotic rectal surgery (*p* = 0.43 and *p* = 0.14, respectively). The MSP values were also comparable at 6 and 12 months after rectal surgery between the two groups (*p* = 0.71 and *p* = 0.71, respectively; Fig. 4B).

## Discussion

The current study evaluated the outcomes of robotic rectal surgery based on the presence of a preexisting stoma. The total operative and console times were prolonged in the S group; however, the other main outcome measures were not affected by the existing stoma. Regarding the impact of a preexisting stoma on minimally invasive abdominal surgery, only a few case reports or case series have investigated how the existence of a stoma influenced laparoscopic bariatric surgery in the literature [27, 28]. In a study of six patients with end ileostomy or colostomy who underwent sleeve gastrectomy or bypass surgery, no conversion to laparotomy and no short-term complications were observed [28]. Conversely, several studies on robotic abdominal surgery have been conducted in patients with a stoma using the ostomy site as a trocar site. For example, the safety and feasibility of robotic Hartmann reversal have often been reported [29, 30]; however, no bowel lifted to the abdominal wall was observed in these study patients because a preexisting stoma was removed at the beginning of surgery. In a similar setting, we previously reported the advantages of laparoscopic resection of the residual rectum as a secondary surgery after total abdominal colectomy for ulcerative colitis over open surgery by placing a port at the ileostomy site [31].

The median console time was prolonged by 114 min in the S group. Suboptimal port placement for the assistant and the existence of a stoma may result in insufficient traction or suction during robotic surgery, thus impeding surgical procedures. Conversely, the difference in operative time did not reach statistical significance. This might be partly explained by the additional time required to create a new diverting stoma after undocking the robot in a substantial number of patients in the NS group.

An imbalance of neoadjuvant treatments was observed between the NS and S groups. Radiotherapy generally causes edematous and fibrotic changes in the mesorectum [32, 33]. These modifications were further intensified in association with the combined use of oxaliplatin-based chemotherapy [32]. High CT density after radiation mirrors inflammatory alterations [33] and may be associated with the difficulty in minimally invasive surgery for rectal cancer as more exudate is generated at the dissection plane and mist is generated [22]. However, the effects of systemic chemotherapy alone are considered mild [34]. Considering the outnumbered patients receiving NACRT in the NS group, the difficulty (long console time) in robotic rectal surgery in association with the presence of a stoma may still be underestimated.

The transverse colon was selected as a stoma site for diversion before the neoadjuvant treatment for obstructive rectal cancer in the current study. Colostomy has several shortcomings, such as frequent stomal prolapse and parastomal hernia and closure difficulty [35, 36]. However, ileostomy cannot resolve colonic distention [13]. Moreover, colostomy is advantageous over ileostomy in terms of a low risk of renal impairment during chemotherapeutic drug administration [37, 38], which we also consider crucial in implementing neoadjuvant treatment. Fortunately, the stoma site for right transverse colostomy usually does not overlap with the typical da Vinci port sites for rectal surgery.

Robotic rectal surgery was started in 2012 at our department as a high-volume center in Japan. The initial model of the robotic platform, da Vinci S, was substituted by da Vinci Xi at the end of 2017. Because perioperative outcomes of robotic rectal surgery were improved by recent models [39, 40], the study patients from 2018 were included when da Vinci Xi was exclusively used.

Exacerbated prolapse of the preexisting stoma was the only remarkable complication observed in the S group in the current study. Several risk factors for stomal prolapse have been identified, including an increase in abdominal pressure, redundant intestine, an oversized stoma route through the abdominal wall, weakness of the abdominal fascia, obesity, advanced age, and chronic obstructive lung diseases [41]. The patient had experienced stomal prolapse before robotic surgery, and persistent pneumoperitoneum for a long operative time may have aggravated the condition. These pathophysiological mechanisms should be considered in laparoscopic or robotic abdominal surgery in patients with a stoma.

Although the difference did not reach a statistical significance, the S group showed a rather favorable RFS even though more patients had advanced stage before neoadjuvant treatments. The paradox may be caused primarily by the small number of patients in the S group. In addition, it may be attributed to the marked down-staging in the S group as evidenced by the similar final stage distribution (Table 4). The responders to neoadjuvant treatments might have had favorable oncological prognosis even if they had distant metastasis. On the other hand, patients with a stoma and distant metastasis might continue to receive systemic therapies without proctectomy unless the disease was deemed easily resectable, and they were not included in the S group; the bias in selecting patients could be another explanation.

Several limitations should be acknowledged in this study. This was a retrospective study conducted at a single institute using a small cohort. The S group comprised only less than ten patients, and fewer patients could be evaluated in the anomanometric data analysis, partly because of the COVID-19 pandemic. These could lead to type II errors in our analyses. In addition, our method of port placement may differ from that at other institutes, which may hinder the generalizability of the current results. For example, if two da Vinci ports are placed in the left upper quadrant instead of one port, more efficient traction of the mobilized segment by the assistant may be allowed avoiding collision between robotic arms and devices used by the assistant. Moreover, different robotic platforms providing a large space around the trocars may achieve better performance by the assistant in patients with a stoma for similar reasons.

In conclusion, robotic sphincter-preserving surgery for rectal cancer in patients with a preexisting stoma is a safe procedure with prolonged console time and does not affect oncological and functional outcomes. Although surgical outcomes were considered acceptable, these patients may require further improvement of minimally invasive surgery.

## Data Availability

The datasets generated during and/or analyzed during this study are available from the corresponding author on reasonable request.
